# Perineural spread evaluation of cranial nerves in nasopharyngeal carcinoma: superiority and reliability of isovolumetric MR imaging

**DOI:** 10.3389/fonc.2024.1492465

**Published:** 2024-11-29

**Authors:** Dechun Zheng, Shugui Xu, Guojing Lai, ChunMiao Hu, Xisheng Cao, Meimei Feng, Li Peng

**Affiliations:** Clinical Oncology School of Fujian Medical University, Fujian Cancer Hospital, Fuzhou, China

**Keywords:** isovolumetric imaging, 3D LAVA_Flex, perineural spread, cranial nerve, nasopharyngeal carcinoma

## Abstract

**Purpose:**

The purpose of this study was to investigate the advantage of three-dimensional liver acquisition with volume acceleration-flexible (3D LAVA_Flex) for perineural spread (PNS) status of nasopharyngeal carcinoma (NPC) in comparison with two-dimensional magnetic resonance sequences.

**Materials and methods:**

Sixty pathological proved NPC patients were prospective enrolled. A protocol included T2-weighted imaging with fat suppression (T2WI fs), T1-weighted imaging (T1WI) without and with contrast enhancement (T1WI ce), and 3D LAVA_Flex was applied for the recruited subject. After determining radiologic diagnostic criteria, the PNS status of cranial nerves (CNs) was carefully interpreted and recorded at the nerve level, anterior and posterior subgroup level, and individual level, respectively. Chi-square test [or McNemar-Bowker (MB) test], Fisher test, and intraclass correlation analysis were used. A *P* < 0.05 indicated statistical significance.

**Results:**

PNS rates of the CNs in the advanced T3 to T4 stage subgroup were significantly different in evaluations performed with 3D LAVA_Flex, T2WI fs, T1WI, and T1WI ce at the patient level (*n* = 51, MB test, all *P* ≤ 0.031) and posterior CN level (MB test, all *P* ≤ 0.016). At the nerve level, 3D LAVA_Flex showed greater PNS detectability than T2WI fs, T1WI, and T1WI ce for CN V3 division (*P* = 0.031, 0.016, and 0.016, respectively), hypoglossal nerve (*P* = 0.002, 0.016, and 0.008, respectively), and external posterior CN IX–XII in carotid space (all *P* = 0.001), and T2WI fs and T1WI for CN IX–XI (*P* = 0.031, 0.001).

**Conclusions:**

3D LAVA_Flex could improve both accuracy and reliability of PNS evaluation of CNs in the advanced NPC cohort and may facilitate decision making for therapeutic strategies.

## Introduction

Pathologically proven perineural invasion (PNI) has been recognized as an independent poor prognostic factor for multiple malignancies (1). However, recent mechanistic studies have suggested that nerve–cancer interactions occur earlier than physical interaction (2). Ligia (2) concluded that PNI showed three different microscopic findings: infiltration, encirclement, or coverage of at least 33% of the nerve by the tumor. Evaluation of perineural spread (PNS), an alternative term that describes neural involvement by primary tumor evidencing by imaging approach, in advanced head and neck cancers at baseline is a strong need for radiologists and physicians for treatment stratification (3) since the pathological approach is not always applicable and shows several other limitations. Thus, there is an urgent need for radiologists to develop and validate a well-proven technique that could give a more reliable PNS assessment at baseline for malignancies.

Magnetic resonance imaging (MRI) can provide valuable TNM (tumor, node, metastasis) staging information in patients with nasopharyngeal carcinoma (NPC). A single-institute study showed that the detection rate of asymptomatic PNS with conventional two-dimensional (2D) MRI sequences was higher than those with evaluation of clinical cranial nerves (CNs) palsy alone (36% vs. 8.9%) and that MRI-evidenced CN involvement was correlated with poor 3-year overall survival and distant metastasis-free survival rates (*P* < 0.002) in the T3-4 NPC subgroup (4). However, the involvement rate of conventional MRI sequences varied among different studies (5, 6). CN palsy is the current standard for evaluating neural invasion for advanced NPC in clinical practice (7), which is supposed to be more biased than MRI. However, the PNS evaluation remains a challenge for MRI with moderate reproductivity and reliability in extracranial head and neck regions. Therefore, an innovative MR technique that could improve confidence, reliability, and repeatability of radiologic findings is warranted for PNS assessment. Efforts to develop a novel non-invasive tool for PNS evaluation have been ongoing for decades since 1985 (8). In comparison with other imaging modalities, MRI is an invaluable technique in terms of its inherent high soft-tissue resolution characteristic; thus, it can be used to detect PNS before the appearance of clinical symptoms (9). In MR neurography, both morphological and functional sequences have shown specific applications for conditions such as congenital, traumatic, vascular, and neoplastic diseases (10, 11). New and evolving sequences have been studied and introduced in clinical practice for investigating the infiltration of the maxillofacial region, skull base, and CNs by multiple head and neck cancers (12, 13). Recently, Kim et al. (14) suggested that a three-dimensional (3D) double-echo steady state with water excitation sequence could improve the visualization of the intra-parotid facial nerve, thus helping surgeons better plan for deep-seated parotid tumors. Contemporary 3D post-gadolinium T1-weighted imaging is increasingly being investigated in neuroimaging and demonstrates superior performance over 2D sequences for neoplastic applications (15). The main pathways and potential spread routes along the CNs for the PNS of head and neck cancers are concluded by researchers (16). The advantages of iso-volumetric 3D imaging sequences include submillimeter evaluation of nerves, fewer artifacts from vessel pulsation (17), an acceptable acquisition time, and detailed anatomic structures that allow examiners to identify and explore the tumor–nerve relationship with multiplanar reconstruction despite the slenderness of the CNs (18). However, there is little knowledge about its advantages in comparison with conventional MRI sequences for evaluating PNS in advanced NPC.

Thus, this study aimed to explore whether isotropic three-dimensional liver acquisition with volume acceleration-flexible (3D LAVA_Flex) could better trace the course of CNs continually and objectively identify PNS in comparison with conventional sequences in patients with NPC.

## Materials and methods

### Patient population

**Figure 1:**
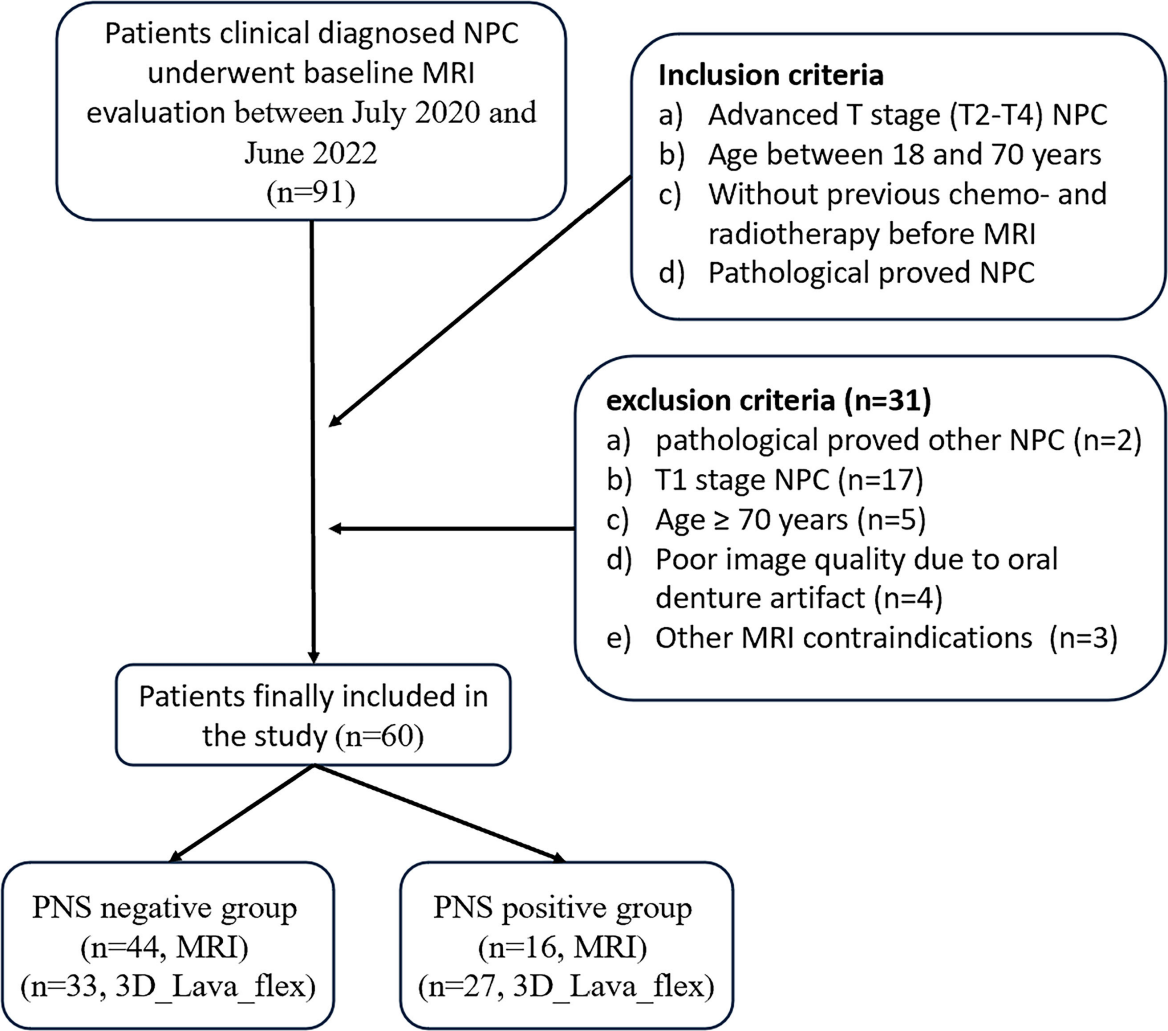
Flowchart of the study enrollment. NPC, nasopharyngeal carcinoma; MRI, magnetic resonance imaging; 3D_Lava_Flex, three-dimensional liver acquisition with volume acceleration-flexible (3D LAVA_Flex).

Ninety-one patients with clinical diagnosed NPC who were referred for baseline regional head and neck MRI evaluations between July 2021 and June 2022 were prospectively enrolled in this study. The primary exclusion criteria were as follows: (a) pathologically proven other diseases; (b) MR-confirmed early T1N0-3M0-1 subjects; (c) over 70 years old; (d) the presence of unremovable metal dentures; and (e) claustrophobia, pregnancy, allergy to gadolinium, and/or other MRI-related contraindications. A total of 31 patients were subsequently excluded (**Figure 1**). The average age of the remaining 60 patients [43 men (71.6%) and 17 women (28.4%)] was 48.5 years (range: 22–66 years). This study was approved by the Institute Committee for Medical and Health Research Ethics (No. 2021-460-01), and written informed consent was obtained from all enrolled patients. Deoxyribonucleic acid of Epstein-Barr virus (EBV-DNA) copy status via plasma EBV-DNA testing was also collected at baseline.

### MRI examination

All patients underwent an MRI scan using a 3.0 Tesla multi-transmit scanner system (Discovery™ MR750w; GE HealthCare, USA) and a 32-channel neurovascular coil. An established NPC staging protocol followed by an additional voluntary MR sequence was performed. The standard NPC staging protocol at our center included axial and coronal plane T2-weighted imaging with fat suppression (T2WI fs); axial and sagittal plane T1-weighted imaging (T1WI); diffusion-weighted imaging (*b* = 0, 800 mm^2^/s); and axial, sagittal, and coronal T1WI contrast enhancement (T1WI ce) with fat suppression after gadolinium administration. Major parameters of T1WI and T2WI sequences: FSE sequences, slice thickness = 5 mm and slice gap = 1 mm for all sequences above.

The isotropic volumetric MR technique used in this study was 3D LAVA_Flex, a gradient-recalled echo sequence. The detailed parameters for this sequence were as follows: repetition time, 13 ms; echo time, 2.6 ms; flip angle, 5°; number of excitations, 4; scan modal, 3D; locs per slab: 256; field of view, 240 mm × 180 mm; acquisition matrix, 160 × 119; vertical resolution, 1 mm; and internal slice resolution, 1 mm ×1 mm. This sequence required an additional scan time of 4 min and 37 s for each enrolled participant.

### Diagnostic criteria and data analysis

**Figure 2:**
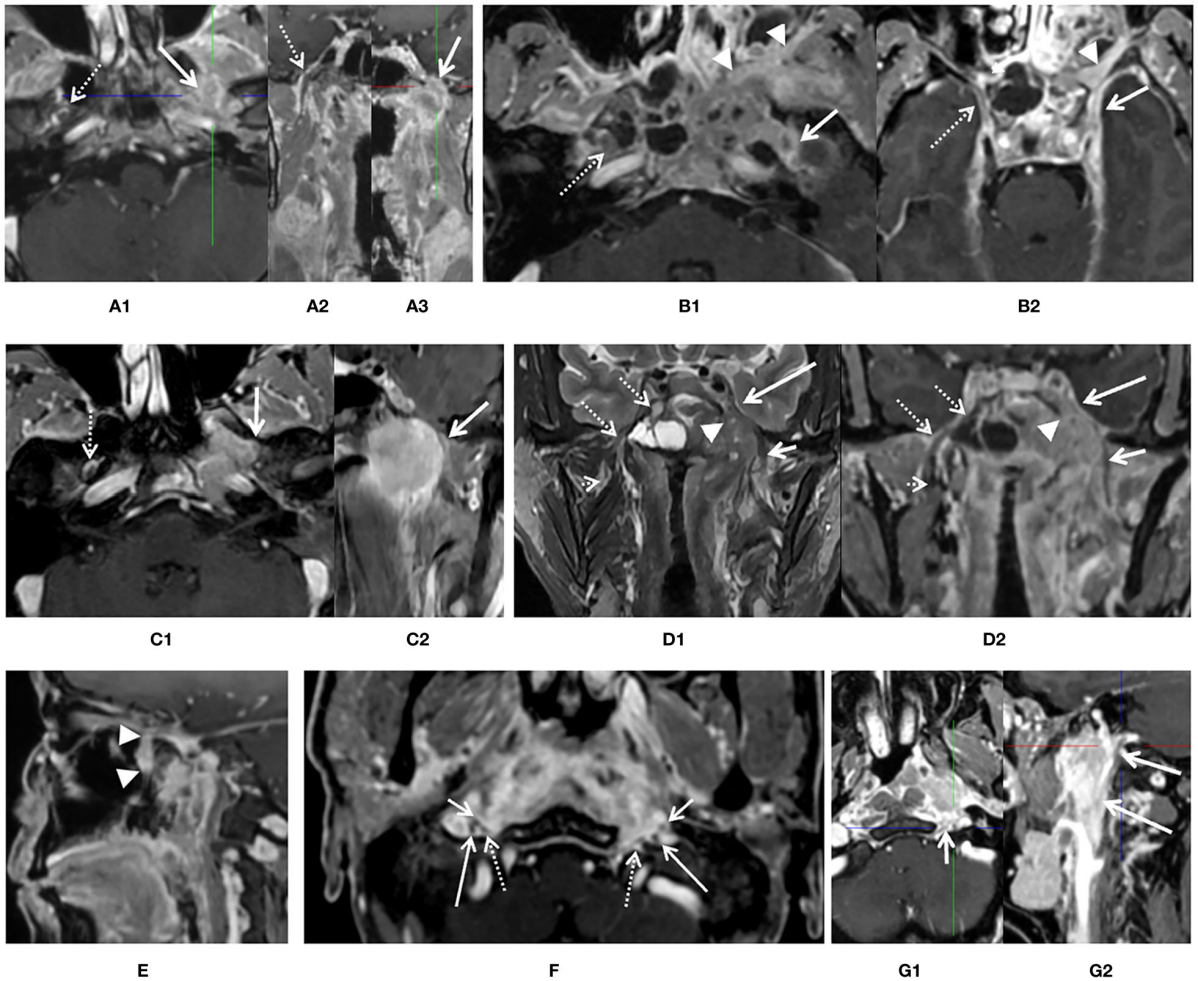
Diagnostic criteria of perineural spread (PNS) by 3D_LAVA_flex on axial, reformatted sagittal, and coronal images. **(A)** An enhancement and enlargement of oval foramen of left side [**(A1, A3)**, white solid arrow] and total disappear of low signal intensity of nerve which suggested an intraneural involvement of mandibular nerve, corresponding normal V3 division [**(A1, A2)**, white dash arrow] was visible on the contralateral side. **(B)** Thickness and enhancement of left oval foramen and rotundum [**(B1, B2)**, solid arrow] suggested PNS positive. Right side is clear (dash arrow). Whole pterygopalatine fossa involvement of left side was also seen (arrow head). **(C)** A thickness and encircle of V3 branch about ½ on left mandibular nerve (solid arrow) suggested infiltration of rotundum segment on axial **(C1)** and reformatted coronal **(C2)** image, right side is normal. **(D)** Whole left Meckel’s cavity (arrow head) and cavernous sinus (long solid arrow) involvement on coronal T2 weighted image **(D1)** and reformatted coronal image **(D2)** was presented and suggested left anterior cranial nerve involvement. Both extracranial and skull base segments of left V3 was also involved (short solid arrow). Meckel’s cavity (dash arrow) and cavernous sinus (dash arrow) on right side is normal. **(E)** Whole pterygopalatine fossa involvement of left side refers to as a sign of maxillary nerve involvement (arrow head). **(F)** At least one third of the extracranial segment of bilateral hypoglossal nerve (dash arrow) and CN IX–XI (long and short arrow) were involved or encircled by primary lesion in the upper carotid space. **(G)** At least one third of the extracranial segment of left hypoglossal nerve (short arrow) and CN IX–XI (long arrow) were involved or encircled by primary lesion in the upper carotid space, while not for right side.

With consideration of prior published standards (2, 13), the following diagnostic findings were applied to define PNS positivity of CNs by primary lesion, as shown in **Figure 2**: (1) intraneural involvement of the nerve and disappearance of normal nerve signal on imaging (**Figure 2A**); (2) thickening of the CN sheath in comparison with the contralateral normal nerve and total encirclement of the nerve by the tumor (**Figures 2B1, B2**); (3) thickening of the CN sheath and coverage of at least one third of the nerve by the tumor with or without displacement (**Figures 2C1, C2, F, G1, G2**); (4) enlargement of the foramen at the skull based on imaging with skeleton erosion (**Figures 2A, C1**); (5) whole pterygopalatine fossa involvement, which served as a sign of maxillary nerve involvement (**Figure 2E**); (6) whole Meckel’s cavity involvement, which served as a sign of trigeminal nerve involvement (**Figures 2D1, D2**); (7) whole cavernous sinus involvement, which served as a sign of anterior CNs involvement (**Figure 2D2**); and (8) whole orbital apex fossa involvement, which served as a sign of optic nerve involvement (not found in any patient in current study), (9) accompany with denervation changes of tongue and masticatory muscle. Absence of any of these findings was defined as PNS negativity.

On the basis of aforesaid criteria, two head and neck radiologists with more than 10 years of experience independently evaluated the status of CN involvement on the basis of T2WI fs, T1WI, T1WI ce, and 3D LAVA_Flex images, respectively. A second evaluation was repeated with a 4-week interval to carry out intra-observer and inter-observer repeatability and reliability. Multiplanar reformation (MPR) was routinely applied for PNS evaluation using the volumetric 3D LAVA_Flex sequence. The PNS status of the 12 pairs of CNs in patients with NPC was assessed. First, locations where primary tumor involved CNs in this cohort were included and named as follows: (1) pterygopalatine fossa and foramen rotundum (maxillary nerve, CN V2), (2) parapharyngeal space and oval foramen (mandibular nerve, CN V3), (3) cavernous sinus (anterior CN III–V), (4) only an involvement of the semilunar sensory ganglion in Meckel’s cave (trigeminal nerve, CN V), (5) hypoglossal canal (hypoglossal nerve, CN XII), (6) jugular foramen region (posterior CN IX–XI), and (7) the carotid space (external posterior CN involvement by primary lesion with/without lymph nodes) for each participant was evaluated. Then, information regarding the PNS status (positivity or negativity), lateral (left, right, or bilateral), and locations was recorded at individual level, anterior (CN I–VI)/posterior (CN VII–XII) subgroup level, and nerve level, respectively, for further analysis.

For all patients’ final PNS status, a consensus was reached after discussion by a third radiologist who has 25 years experiences on head and neck region whenever there was any conflict between the two radiologists. Our patients showed no orbital apex or internal auditory canal involvement. Therefore, data for the PNS involvement of the external anterior CN I to CN VI (ophthalmic nerve) and posterior CN VII and CN VIII were unavailable.

### Statistical analysis

Data were analyzed using SPSS software (version 19.0; SPSS, Chicago, IL, USA). The CN involvement status was stratified into individual, anterior and posterior subgroups, and nerve levels. The ability and power of these four different sequences to diagnose PNS in advanced T3-4 NPC patients at the three rank levels were, respectively, evaluated using the paired chi-square test and McNemar-Bowker (MB) test. The advantage of using the volumetric sequence for PNS detection was determined by comparing the original T stage and modified T stage on the basis of additional positive findings on 3D LAVA_Flex images using the chi-square test. The correlation between PNS status and EBV-DNA was evaluated using Pearson correlation. Intraclass correlation coefficient (ICC) was used for intra-observer and inter-observer repeatability and reliability evaluation. Significance was set at *P* < 0.05.

## Results

### Patient characteristics

A total of 60 patients with NPC participated in this pilot study between July 2020 and November 2021, including 43 male and 17 female patients. The mean age in this group was 48.57 ± 10.35 years (range: 22–66 years). Demographic characteristics of the study population are shown in **Table 1**. Among the 27 patients with PNS (T4) determined by conventional MR and 3D LAVA_Flex sequences, only four patients had proven clinical symptoms and signs (three with detectable numbness along trigeminal branches and the other one with his tongue title to the left when stretching out during physical exam), while the other 23 did not. None of these 27 patients presented denervation changes. 3D LAVA_Flex and MPR indicated additional asymptomatic CN PNS in 10 of 35 T3-stage and 1 of 9 T2-stage NPC patients compared to conventional MR, who could be upstaged from T2-3 to T4 stage accordingly. In brief, 3D LAVA_Flex witness a significant higher PNS ratio than conventional MR in this study (27 vs. 16, *P* < 0.001). The EBV-DNA positive ratio was 93.3% (56/60).

**Table 1:** Patient demographics and clinical characteristics.

Characteristics	Total (*n* = 60)	Total (*n* = 60)
Age, years		
median (range)	48 (22–66)	48 (22–66)
Sex		
Male	43	–
Female	17	–
Histopathologic type		
Non-keratinized undifferentiated SCC	57	–
Keratinized undifferentiated SCC	1	–
Poorly differentiated SCC	2	–
EBV-DNA status		
Positive	56	56
Negative	4	4
T stage	(without 3D LAVA_Flex)	(with 3D LAVA_Flex)
T2	9	8
T3	35	25
T4	16	27
N stage		
N0	4	–
N1	25	–
N2	17	–
N3	14	–
M stage		
M0	51	–
M1	9	–
AJCC/UICC stage	(without 3D LAVA_Flex)	(with 3D LAVA_Flex)
Stage II	4	4
Stage III	27	16
Stage IVA	20	31
Stage IVB	9	9

SCC, squamous cell carcinoma; 3D LAVA_Flex, three-dimensional liver acquisition with flexible volume acceleration; EBV, Epstein-Barr virus.

### PNS ratio comparison between 3D LAVA_Flex and three conventional sequences

Statistical analysis was performed at three levels (individual level, anterior/posterior CN subgroup level, and nerve level) to investigate statistically significant differences between the 3D LAVA_Flex and conventional sequences in evaluating the PNS rate in the advanced T3 to T4 NPC subgroup (*n* = 51); details are provided in **Table 2** (3D LAVA_Flex vs. T2WI fs), **Table 3** (3D LAVA_Flex vs. T1WI), and **Table 4** (3D LAVA_Flex vs. T1WI ce).

**Table 2:** PNS detection comparison between 3D LAVA_Flex and T2WI fs in T3 and T4 stage nasopharyngeal carcinoma.

Level		T2WI fs 3D LAVA_Flex		T2WI fs		Total	MB test
	Nerve			negative	positive		*P* value
1	mandibular nerve		negative	35	0	35	0.031
			positive	5	11	16	
		Total		40	11	51	
1	maxillary nerve		negative	40	0	40	0.125
			positive	3	8	11	
		Total		43	8	51	
1	cavernous sinus (CN III–V)		negative	40	0	40	0.500
			positive	1	10	11	
		Total		41	10	51	
2	anterior CNs		negative	25	0	25	0.063
			positive	4	22	26	
		Total		29	22	51	
1	hypoglossal nerve		negative	37	0	37	0.002
			positive	9	5	14	
		Total		46	5	51	
1	jugular foramen (IX–XI nerves)		negative	44	0	44	0.031
			positive	5	2	7	
		Total		49	2	51	
1	carotid space (IX–XII, primary lesion and/or lymph nodes)		negative	25	0	25	0.001
			positive	19	7	26	
		Total		44	7	51	
2	posterior CNs		negative	37	0	37	0.002
			positive	9	5	14	
		Total		46	5	51	
3	individual level (all above)		negative	22	0	22	0.001
			positive	7	22	29	
		Total		29	22	51	

PNS, perineural spread; CN, cranial nerve; 3D LAVA_Flex, three-dimensional liver acquisition with volume acceleration-flexible; fs, fat suppression; MB, McNemar Bowker.

**Table 3:** PNS detection comparison between 3D LAVA_Flex and T1WI in T3 and T4 stage nasopharyngeal carcinoma.

Level		T1WI 3D LAVA_Flex		T1WI		Total	MB test
	Nerve			negative	positive		*P-*value
1	mandibular nerve		negative	35	0	35	0.016
			positive	6	10	16	
		Total		41	10	51	
1	maxillary nerve		negative	40	0	40	0.125
			positive	3	8	11	
		Total		43	8	51	
1	cavernous sinus (CN III–V)		negative	40	0	40	0.063
			positive	4	7	11	
		Total		44	7	51	
2	anterior CNs		negative	25	0	25	0.031
			positive	5	21	26	
		Total		30	21	51	
1	hypoglossal nerve		negative	37	0	37	0.016
			positive	10	4	14	
		Total		47	4	51	
1	jugular foramen (IX–XI nerves)		negative	44	0	44	0.001
			positive	6	1	7	
		Total		50	1	51	
1	carotid space (IX–XII, primary lesion and/or lymph nodes)		negative	25	0	25	0.001
			positive	21	5	26	
		Total		46	5	51	
2	posterior CNs		negative	37	0	37	0.001
			positive	10	4	14	
		Total		47	4	51	
3	individual level (all above)		negative	22	0	22	0.008
			positive	7	22	29	
		Total		29	22	51	

PNS, perineural spread; CN, cranial nerve; 3D LAVA_Flex, three-dimensional liver acquisition with volume acceleration-flexible; MB, McNemar Bowker.

**Table 4:** PNS detection comparison between 3D LAVA_Flex and T1WI ce in T3 and T4 stage nasopharyngeal carcinoma.

Level		T1WI ce 3D LAVA_Flex		T1WI ce		Total	MB test
	Nerve			negative	positive		*P-*value
1	mandibular nerve		negative	35	0	35	0.016
			positive	6	10	16	
		Total		41	10	51	
1	maxillary nerve		negative	40	0	40	0.125
			positive	3	8	11	
		Total		43	8	51	
1	cavernous sinus (CN III–V)		negative	40	0	40	0.500
			positive	1	10	11	
		Total		41	10	51	
2	anterior CNs		negative	25	0	25	0.063
			positive	4	22	26	
		Total		29	22	51	
1	hypoglossal nerve		negative	37	0	37	0.008
			positive	7	7	14	
		Total		44	7	51	
1	jugular foramen (IX–XI nerves)		negative	44	0	44	0.125
			positive	3	4	7	
		Total		47	4	51	
1	carotid space (IX–XII, primary lesion and/or lymph nodes)		negative	25	0	25	0.001
			positive	17	9	26	
		Total		42	9	51	
2	posterior CNs		negative	37	0	37	0.016
			positive	6	8	14	
		Total		43	8	51	
3	individual level (all above)		negative	22	0	22	0.031
			positive	5	24	29	
		Total		27	24	51	

PNS, perineural spread; CN, cranial nerve; 3D LAVA_Flex, three-dimensional liver acquisition with volume acceleration-flexible; CE, contrast enhancement; MB, McNemar Bowker.

**Figure 3:**
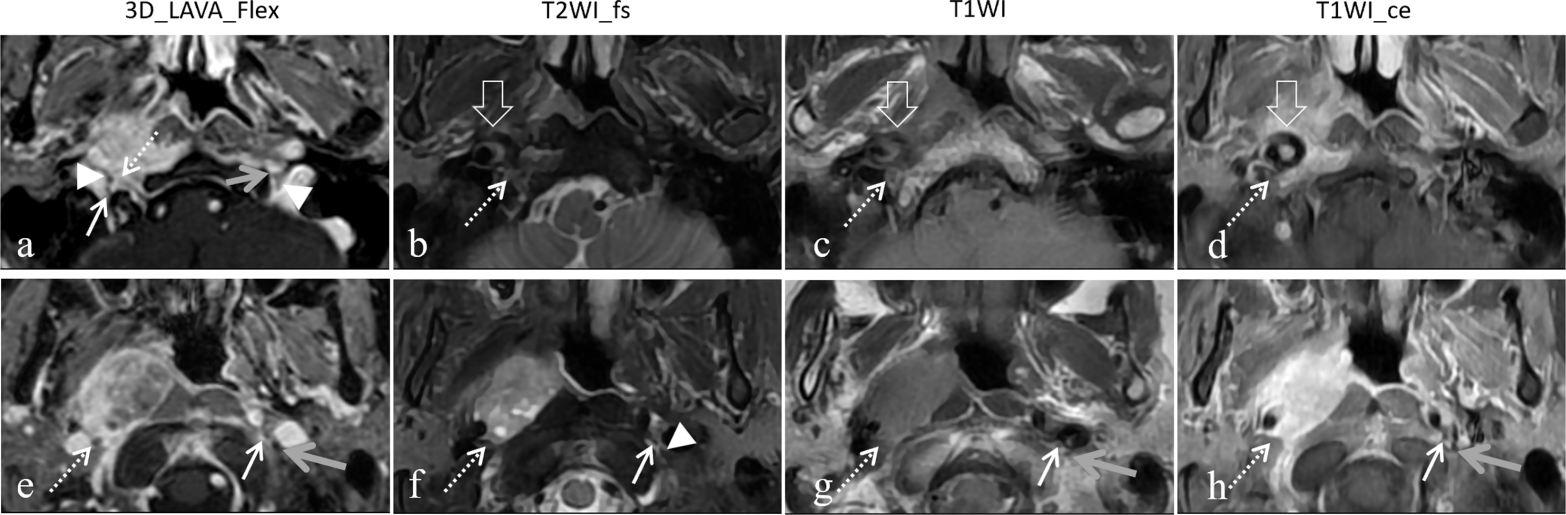
Representative images showed the different capacity to determine cranial nerve status between 3D LAVA_Flex and conventional sequences. A 54-year-old female nasopharyngeal carcinoma patient with right posterior cranial nerves involvement from the primary lesion and retropharyngeal lymph node (RPN). The hypoglossal nerve was infiltrated by at least one-third encirclement (dashed arrow) on 3D LAVA_Flex while showing normal findings on the left side (gray arrow), and the bilateral vagus nerve (arrowhead) and right accessory nerve (solid arrow) can be seen **(A)**. This was also at least one-third involvement for the vagus nerve (dashed arrow), which suggested infiltration from the RPN 20 mm below it **(E)**. No positive PNI imaging signs from the primary lesion and RPN were observed on T2WI fs **(B, F)**, T1WI **(C, G)**, and T1WI ce **(D, H)** images. Some artifacts were observed on the T2WI fs, T1WI, and T1WI ce images.

At the individual level, statistically significant differences were observed in the evaluation of PNS of the CN in advanced T3 to T4 stage subjects by 3D LAVA_Flex and T2WI fs, T1WI, or T1WI ce (MB test, *P ≤* 0.031 Level 3). Furthermore, statistically significant differences were also observed in evaluations at the posterior CN level between 3D LAVA_Flex and the three conventional sequences (MB test, all *P ≤* 0.016, Level 2), and at the anterior CN level between 3D LAVA_Flex and T1WI (*P* = 0.031), but not at the anterior CN level (T2WI fs and T1WI_CE, *P* = 0.063). 3D LAVA_Flex helped identifying PNS of CN in another 11 patients who showed negative imaging findings on T2WI fs, T1WI, or T1WI ce sequences (**Figure 3**). Among these 11 patients, 3 and 8 presented with anterior or posterior CN involvement, respectively.

The MB test also showed statistically significant differences in the identification of PNS for NPC staging at the nerve level between 3D LAVA_Flex and the conventional sequences, as detailed in **Tables 2** **–** **4** (Level 1). In brief, the 3D LAVA_Flex sequence was able to determine more cases of PNS than T2WI fs, T1WI, and T1WI ce for the mandibular nerve (*P* = 0.031, 0.016, and 0.016, respectively); T2WI fs, T1WI, and T1WI ce for the hypoglossal nerve (*P* = 0.002, 016, 008, respectively); T2WI fs and T1WI for posterior CN IX–XI (*P* = 0.031, 0.001); and T2WI fs, T1WI, and T1WI ce for external posterior CN IX–XII involvement by primary lesion with or without lymph node fusion (all *P* = 0.001). The findings also indicated a statistically significant difference in determining PNS status, which referred to the T4 stage, between 3D LAVA_Flex and conventional sequences (31 vs. 20, *P* = 0.001, Fisher test). However, no statistically significant difference (*P* > 0.05) was observed between T2WI fs, T1WI, and T1WI ce for evaluating PNS in NPC.

### Repeatability of PNS status evaluation of four different sequences

The interpretation consistency of PNS through four different sequences was investigated through agreement analysis. As shown in **Table 5**, the intra-observer and inter-observer coefficients (*n* = 56 in all) of T2WI fs, T1WI, T1WI ce, and 3D LAVA_Flex in determining PNS status were favorable (29/56) or excellent (19/56) in major situations (ICC ≥ 0.75), except an 8/56 (14.3%) indicated a ICC ranged between 0.565 and 0.715. All of eight occurred at conventional sequences.

**Table 5:** The analysis of intra- and inter-observer reproducibility.

	Intra-observer				Inter-observer			
	T2WI fs	T1WI	T1WI ce	3D LAVA_Flex	T2WI fs	T1WI	T1WI ce	3D LAVA_Flex
**Individual level**	0.790	0.777	0.777	0.865	0.904	0.852	0.821	0.918
Nerve level								
mandibular nerve	0.684	0.695	0.565	0.781	0.897	0.882	0.753	0.879
maxillary nerve	0.874	0.874	0.797	0.759	0.959	0.874	0.869	0.933
cavernous sinus	0.865	0.699	0.887	0.813	0.934	0.773	0.969	0.861
hypoglossal nerve	0.805	0.843	0.876	0.962	0.986	0.984	0.986	0.991
jugular foramen (IX–XI nerves)	1.000	1.000	0.869	0.929	1.000	1.000	0.869	1.000
carotid space (IXx–XII nerves)	0.791	0.581	0.854	0.789	0.715	0.632	0.639	0.794

3D LAVA_Flex, three-dimensional liver acquisition with volume acceleration-flexible; CE, contrast enhancement.

## Discussion

This preliminary study suggested that the new isotropic volumetric 3D LAVA_Flex sequence offered an advantage in determining PNS status in NPC staging in comparison with conventional sequences in T3 and T4 NPC patients (*n* = 51), at the individual level, anterior and posterior CN subgroup level, and nerve level (all *P* < 0.05). The 3D LAVA_Flex sequence also helped identify more asymptomatic PNS-positive cases, a total of 1 T2 stage and 10 T3 stage NPC subjects (8 of 11 with posterior CN positivity) were supposed to be modified as T4 stage with a combination of volumetric techniques.

Both CT and MRI are useful for depicting the CN course and concluding a PNS diagnosis for head and neck cancers (19). Some T2WI-based sequences have been used to examine the intracranial segment of CNs and the related diseases (20). However, when referring to the tumor setting, T1WI-based sequences are supposed to offer an advantage in depicting the in-detail relationship and invasive status with tumors, especially when evaluating skull base and extracranial segments (20). As for head and neck cancers, the estimated PNI or PNS rate was reported to be 27% to 82%, and PNS was reported to be more frequent in adenoid cystic carcinoma (up to 96%) (16, 21, 22). Although more than 40% of patients with PNS are asymptomatic, MRI is the preferred imaging modality for PNS because of its inherent advantages of soft tissue contrast, especially on a 3T unit (23, 24). NPC is the most common head and neck cancer in China, with a PNS rate of approximately 8.0% to 12.4% (clinically symptomatic) or 60.8% (MR imaging modality) because of its special biological behavior (6, 25).

For decades, both physicians and radiologists have attempted to improve the accuracy of TNM classification and PNS status evaluation for regions such as bone structures, the parapharyngeal space, masticator space, carotid space, and visceral space. The 3D LAVA_Flex technique available in a clinical scanner, which uses a block data acquisition pattern, could offer a thinner slice thickness (0.7–1.5 mm) without a gap between slices and an isotropic 3D dataset for arbitrarily multiplanar reconstruction whenever needed during imaging interpretation and could improve an in-depth insight to the earlier minor PNS invasion than conventional 2D sequences. A recent pilot study evaluated 37 NPC patients with pterygopalatine fossa (PPF) involvement and suggested that the 3D volumetric MR technique could help depict PNS of the invaded PPFs and its connecting conduits (vidian canal, palatovaginal canal, and sphenopalatine foramen) (25). These findings provided exciting information regarding the ability of 3D volumetric MR imaging to characterize external CN involvement by head and neck tumors such as NPC. Our study results were comparable with this finding, since 3D LAVA_Flex identified PNS-positive results in an additional 10 patients from the 35 T3 subgroup and showed significant differences from the other three conventional sequences (*P* < 0.05). The 3D volumetric MR imaging technique apparently involves fewer magnetic susceptibility artifacts and pulsatile artifacts in the jugular region. Thus, it can clearly depict the course of CNs and their corresponding relationships with primary head and neck tumors.

Other techniques can also help determine PNS status in the head and neck region. Diffusion tensor imaging (DTI) has been suggested to provide quantitative markers, including fractional anisotropy (FA) and apparent diffusion coefficient (ADC), that can help confirm mandibular nerve involvement (14). Another promising technique, [^68^Ga] Ga-labeled fibroblast activation protein inhibitor (^68^Ga-FAPI) positron emission tomography/computed tomography (PET/CT), has been the subject of a number of studies. A recent study suggested that it outperformed ^18^F-FDG PET-CT in the assessment of both primary and local recurrent NPC and showed higher radiotracer uptake in primary lesions, lymph nodes, bone lesions, and visceral metastases, allowing the determination of more true-positive local relapse lesions (26). Zheng et al. (27) conducted a comparable study of PET-CT using two different biomarkers to explore the value of ^68^Ga-FAPI PET/CT in primary NPC patients, and the results were encouraging. ^68^Ga-FAPI PET/CT outperformed ^18^F-FDG PET-CT for the assessment of bone structure involvement (207 vs. 177) and cavernous sinus involvement (10 vs. 1) and served as a useful complement to MRI by upgrading the T stage in 13 patients and underestimating it in two patients in their cohort.

However, our study had several limitations. First, this study did not have a gold standard for affirming PNS. Our diagnostic criteria were the expert consensus, which is clinically used when the pathological standard is unavailable (28). False positive and false negative remain unclear since there is absence of histological criteria of PNS in NPC. Second, the relationship between carotid space involvement (CSI) and PNS from the four posterior CN IX–XII has not been described in the literature. Thus, this primary result requires further evaluation to determine its prognostic value. We noticed that the prognostic value of CSI for NPC has been studied and proven recently with stratification by induction chemotherapy (ICT) (29). In the non-ICT group, CSI was an independent prognostic factor for overall survival (OS), and in the ICT group, OS was improved by ICT with a hazard ratio of 0.41. Finally, our study focused on the diagnostic value of PNS evaluation among the four MR sequences. We did not evaluate the potential influence of the modified T stage on patient’s prognosis. Thus, a single sequence PNS positive and its clinical contribution of 3D LAVA_Flex require further evaluation. Other aspects of this new technique, such as cost-effective analyses, operational complexity, and the new diagnostic interpretation issue, warrant to be studied and resolved before it can generalize in clinic.

In conclusion, 3D_LAVA_flex could provide more reliable evidence about the PNS status in patients with advanced NPC, making it a preferred candidate for NPC scheduled staging protocols, especially in epidemic areas and posterior CN evaluation. The 3D volumetric MR technique would upstage approximately 18.3% of the cohort by redefining the T classification in comparison with the classification performed using conventional sequences alone.

## Data Availability

The raw data supporting the conclusions of this article will be made available by the authors, without undue reservation.
